# Fatty acid synthase is a primary target of MiR-15a and MiR-16-1 in breast cancer

**DOI:** 10.18632/oncotarget.12479

**Published:** 2016-10-05

**Authors:** Jingxuan Wang, Xiao Zhang, Jinming Shi, Paul Cao, Meimei Wan, Qiang Zhang, Yunxuan Wang, Steven J. Kridel, Wennuan Liu, Jianfeng Xu, Qingyuan Zhang, Guangchao Sui

**Affiliations:** ^1^ Department of Medical Oncology, the Third Affiliated Hospital of Harbin Medical University, Harbin P. R. China; ^2^ College of Life Science, Northeast Forestry University, Harbin, China; ^3^ Department of Cancer Biology and Comprehensive Cancer Center; ^4^ Center for Cancer Genomics, Wake Forest University School of Medicine, Winston-Salem

**Keywords:** Fatty acid synthase (FASN), miR-15a and miR-16-1, 3′-UTR, breast cancer, gene expression

## Abstract

Fatty acid synthase (FASN) is upregulated in breast cancer and correlates with poor prognosis. FASN contributes to mammary oncogenesis and serves as a bona fide target in cancer therapies. MicroRNAs inhibit gene expression through blocking mRNA translation or promoting mRNA degradation by targeting their 3′-UTRs. We identified four microRNAs in two microRNA clusters miR-15a-16-1 and miR-497-195 that share a common seed sequence to target the 3′-UTR of the FASN mRNA. In reporter assays, both of these microRNA clusters inhibited the expression of a reporter construct containing the FASN 3′-UTR. However, only ectopic miR-15a-16-1, but not miR-497-195, markedly reduced the levels of endogenous FASN in breast cancer cells. Both miR-15a and miR-16-1 contributes to inhibiting FASN expression and breast cancer cell proliferation. Consistently, a sponge construct consisting of eight repeats of the FASN 3′-UTR region targeted by these microRNAs could markedly increase endogenous FASN levels in mammary cells. When FASN expression was restored by ectopic expression in breast cancer cells, retarded cell proliferation caused by miR-15a-16-1 was partially rescued. In conclusion, we demonstrated that FASN expression is primarily downregulated by miR-15a and miR-16-1 in mammary cells and FASN is one of the major targets of these two tumor suppressive microRNAs.

## INTRODUCTION

In mammalian cells, fatty acid synthase (FASN) is the central lipogenic enzyme promoting the *de novo* synthesis of long-chain fatty acids from acetyl-CoA and malonyl-CoA [1]. Normal cells express very low levels of FASN; however, FASN overexpression has been observed in many types of tumors and correlated with poor prognoses of patients [2–6]. Consistently, inhibition of FASN activity leads to cytotoxic effects, including cell growth arrest and apoptosis [7, 8]. Mechanistically, overexpressed FASN promotes HER2-regulated signaling and leads to enhanced cell proliferation [9, 10]. Recent studies also indicated that FASN plays an important role in regulating expression of genes involved in apoptosis and DNA repair [11]. Thus, inhibiting FASN expression or its activity represents a promising approach of therapeutic treatment for multiple cancers, including breast cancer [2].

MicroRNAs (miRNAs) are small RNAs of 17–24 nucleotides regulating gene expression at the post-transcriptional level. Each miRNA has a seed sequence close to its 5′ end consisting of 7–8 nucleotides that perfectly match the 3′ end of its target site on the 3′-UTR of a mRNA. The binding of a miRNA to its target causes translational inhibition or target mRNA degradation. Each miRNA can theoretically target hundreds of genes [12] and about 30% of human protein coding genes may be regulated by miRNAs [13, 14]. Studies from the last decade indicate that miRNAs play an important role in oncogenesis through downregulating expression of gene regulating cancer development and progression [15]. Whether a miRNA plays an oncogenic or tumor suppressive role depends on the activity of its regulated genes. Additionally, different cancers possess characteristic miRNA expression profiles, which can be potentially used in cancer diagnosis and prognosis [16, 17].

Many miRNAs possess tumor suppressive function. Among them, miR-15a and miR-16-1 have the same seed sequence and exert their tumor suppressive activities through inhibiting multiple proliferative or oncogenic genes, such as Bcl-2, cyclin D1 and WNT3A [18]. These two miRNAs are coded by the two close loci on chromosomal region 13q14 that is frequently deleted in cancer [19]. In addition, the proto-oncogene c-Myc represses miR-15a/miR-16-1 expression [20]. Another two miRNAs, miR-497 and miR-195, are coded by two close loci on chromosome 17. Previous studies indicated that they are downregulated in breast cancer through DNA methylation [21] and their tumor suppressive role has also been suggested in breast and colon cancers [21, 22]. Interestingly, these four aforementioned miRNAs share the same seed sequence, suggesting that they may have common target genes. Multiple reports revealed that FASN expression can be inhibited by different miRNAs including miR-142-3p, miR-320, miR-424 and miR-195 [23–26]. However, all these studies were conducted in osteosarcoma cells and which of them plays the most important role in regulating FASN expression in breast cancer remains unclear. Looking through FASN 3′-UTR, we discovered a stretch that can be targeted by miR-15a-16-1 and miR-497-195. In this study, we show that both miR-15a-16-1 and miR-497-195 clusters blocked the luciferase reporter expression mediated by the FASN 3′-UTR; however, only miR-15a-16-1, but not miR-497-195, inhibits endogenous FASN expression. Importantly, our data strongly suggest that FASN downregulation in mammary cells is primarily achieved by miR-15a-16-1 through targeting their binding site on the FASN 3′-UTR.

## RESULTS

### The miR-15a-16-1 and miR-497-195 clusters and the putative binding site on the FASN 3′-UTR

**Figure 1:**
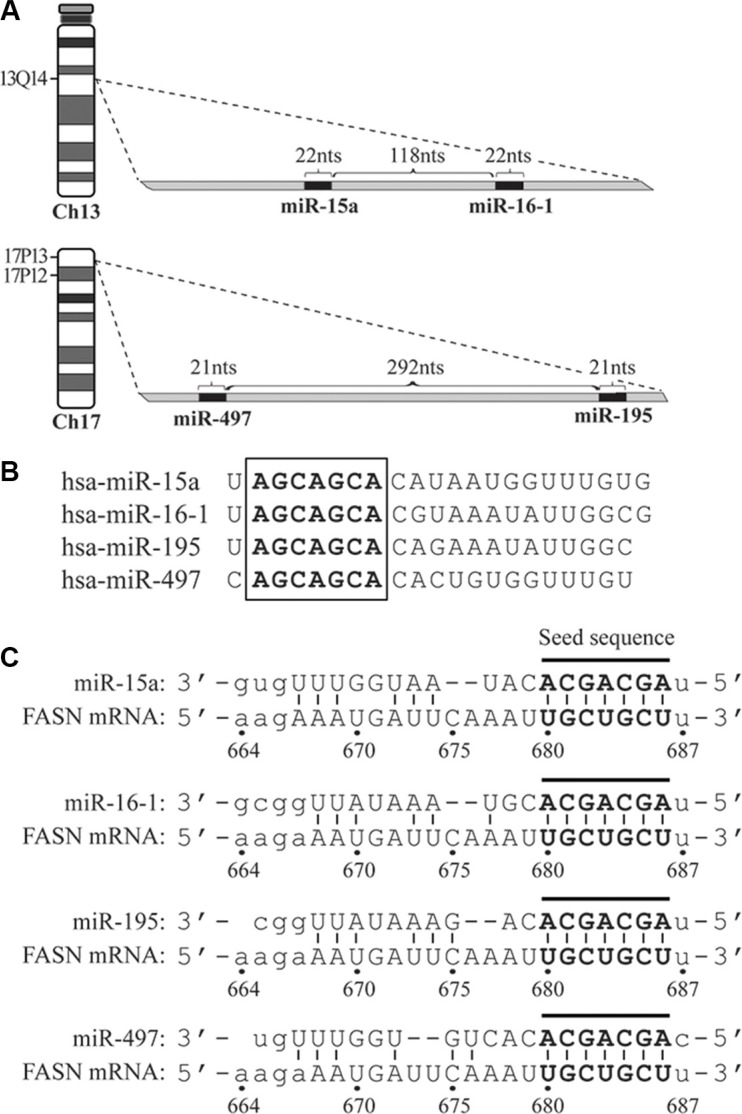
The schematic diagrams of genomic loci for microRNAs, their seed sequences and target site on the 3′-UTR of FASN mRNA (**A**) Genomic loci of miR-15a-16-1 and miR-497-195 on chromosomes 13 and 17, respectively. (**B**) The seed sequences (in bold and embraced by a square) of miR-15a, miR-16-1, miR-497 and miR-195. (**C**) The target site of the four microRNAs on the FASN 3′-UTR. The matched nucleotides between microRNA seed sequences and target site are in bold and connected by short lines.

The miR-15a-16-1 cluster is located at the chromosomal 13q14, close to the tumor suppressor Rb1. The coding regions of miR-15a and miR-16-1 are separated by 118 nucleotides (nts). The miR-497-195 cluster is located at the chromosomal 17p13. MiR-195 and miR-497 are separated by 292 nts (Figure 1A). These four miRNAs share the same seed sequence of 7 nts (5′-AGCAGCA-3′; Figure 1B).

The 3′-UTR of the human FASN mRNA contains 828 nts. To determine miRNAs that potentially block FASN expression, we analyzed FASN 3′-UTR sequence using an algorithm available from the microRNA.org (see [27] and www.microrna.org) to predict potential miRNA target sites. Among the miRNA candidates identified by this algorithm, we found that miR-15a, -16-1, -497 and -195 have their seed sequence reverse-complementarily match a 7-nt stretch (5′-UGCUGCU-3′) on the 3′-UTR of the FASN mRNA. This region is numbered as the 680-686 nts, if we arbitrarily designate the nucleotide right after the FASN mRNA stop codon as “1” and its downstream side as “+” (Figure 1C).

### miR-15a-16-1 and miR-497-195 inhibit FASN 3′-UTR-mediated expression in reporter assays

**Figure 2:**
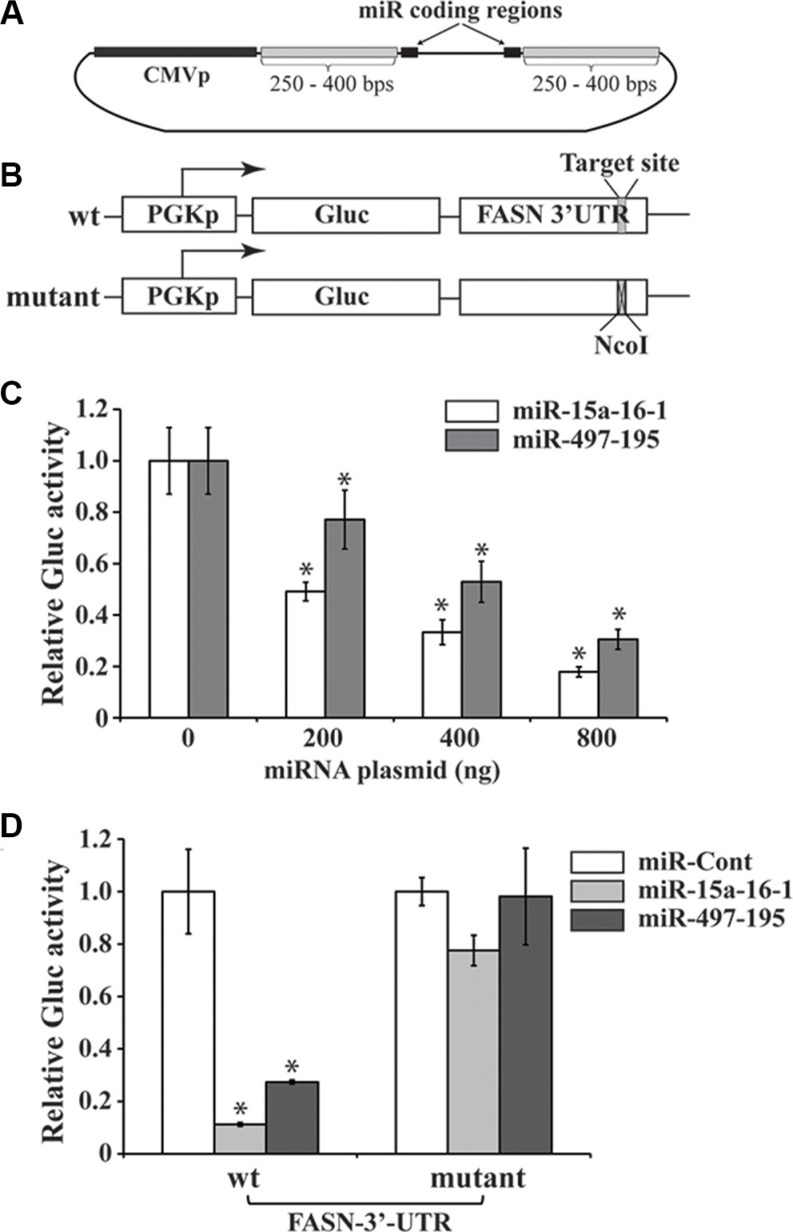
Reporter assays of FASN 3′-UTR-mediated expression regulated by microRNAs (**A**) A schematic diagram of microRNA expressing plasmids. The constructs were generated using the genomic DNA sequence based on a previously reported protocol [40]. The pSL4 lentiviral vector with the CMV promoter to drive microRNA expression and a puromycin antibiotic selection marker is used to express the two microRNA clusters. (**B**) Schematic diagrams of reporter constructs. The wild type (wt) reporter vector, pPGK-Gluc-FASN-3UTR, consists of the PGK promoter, Gluc cDNA and FASN 3′-UTR. The target site of the miR-15a/miR-16-1 seed sequence is indicated. In the mutant reporter construct, 6 bps in this target site was replaced by a NcoI site. (**C**) Reporter assays to test the effects of miRNAs on the Gluc expression mediated by the FASN 3′-UTR. Increasing miR-15a-16-1 and miR-497-195 expression vectors (0, 200, 400 and 800 ng), compensated with a control miRNA vector to 800 ng, if necessary) were co-transfected with wt pPGK-Gluc-FASN-3UTR reporter (50 ng) presented in “A” and the SEAP-expressing plasmid (100 ng) into MCF-7 cells (triplicated). Gluc activity was determined and normalized by SEAP activity (see Materials and Methods for details). (**D**) To determine the importance of miRNA binding site on the FASN 3′-UTR in miRNA-mediated reporter expression. One hundred ng of miR-Cont (i.e. miR-m15a-m16-1), miR-15a-16-1 or miR-497-195 was co-transfected with 50 ng of the wt or mutant reporter construct (50 ng) and the SEAP-expressing plasmid (100 ng). Gluc activity was measured and normalized as described above. Results for C and D are presented as a mean of three observations ± S.D. Statistical significance is shown by *indicating a *p* value < 0.05.

To express these miRNAs, we generated two constructs containing the genomic loci of the miR-15a-16-1 and miR-497-195 miRNAs (Figure 2A), and control constructs expressing mutated miRNAs. To test the effect of these miRNAs on the FASN 3′-UTR mediated expression by reporter assays, we generated two reporter constructs; one contains the wild type (wt) sequence of the FASN 3′-UTR and the other contains this UTR with the putative binding site for the seed sequence of these miRNAs replaced by a *Nco*I site (Figure 2B). We first used the wt construct to co-transfect with different amounts of miR-15a-16-1 or miR-497-195 plasmids. Both miRNA clusters significantly reduced the Gluc expression of the FASN 3′-UTR-containing reporter in a dose-dependent manner (Figure 2C). To determine whether the predicted seed sequence on the FASN 3′-UTR is essential to this inhibition, we also individually used the wt and mutant reporter constructs to co-transfect with the same amount of miR-Cont, miR-15a-16-1 and miR-497-195 constructs, respectively. While both miR-15a-16-1 and miR-497-195 constructs could markedly reduce Gluc expression of the wt reporter compared to the miR-Cont, they did not significantly change the expression of the mutant reporter (Figure 2D). These data suggest that miR-15a-16-1 and miR-497-195 inhibit FASN mRNA expression through targeting the 680-686 nts in the FASN 3′-UTR.

### miR-15a-16-1, but not miR-497-195, inhibits endogenous FASN expression and breast cancer cell proliferation

**Figure 3:**
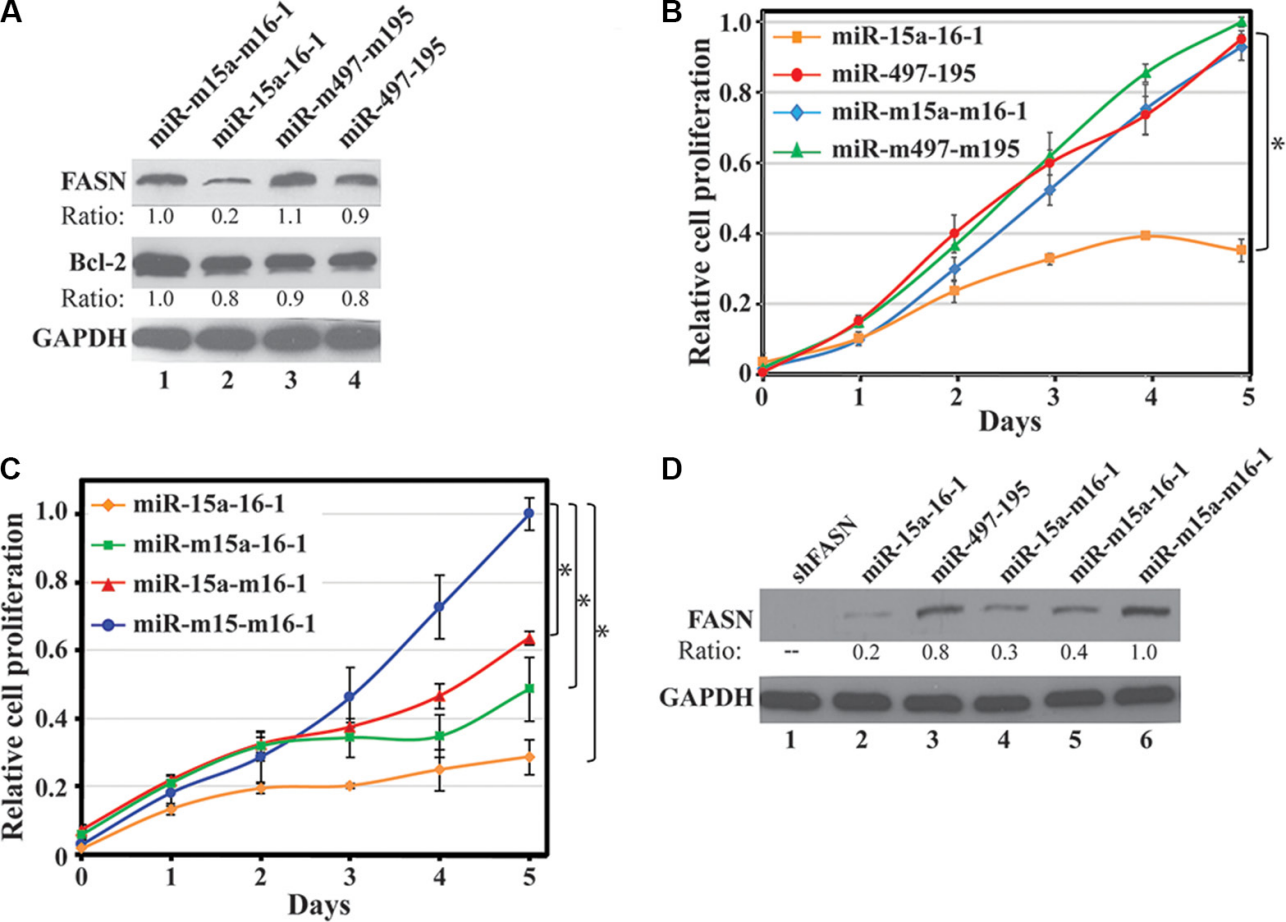
Effect of miR-15a-16-1 and miR-497-195 on endogenous FASN expression and breast cancer cell proliferation (**A**) and (**B**) Effects of ectopic miR-15a-16-1 and miR-297-195 on endogenous FASN and Bcl-2 expression and cell proliferation. MDA-MB-231 cells were individually infected by pSL4 vector-based lentiviruses expressing different miRNAs as labeled. Two days post infection, the cells were cultured in medium containing 1.0 μg/ml of puromycin for 3 days and then collected for Western blot analyses (A) and WST-1 assays (B). (**C**) and (**D**) Effects of ectopic miR-15a and miR-16-1 on cell proliferation and endogenous FASN expression. Experiments similar to A and B were performed for individually infected MDA-MB-231 cells by lentiviruses expressing miR-15a-16-1, miR-m15a-16-1, miR-15a-m16-1 and miR-m15a-m16-1. The treated cells were subject to WST-1 cell proliferation assays (C) and Western blot analyses for FASN expression (D). Results for B and C are presented as a mean of three observations ± S.D. Statistical significance is shown by *indicating a *p* value < 0.05.

We previously reported that a miRNA reduced the expression of a reporter construct but showed no effect on the cognate endogenous gene [28]. Thus, we tested whether miR-15a-16-1 and miR-497-195 also inhibit endogenous FASN protein expression. We used lentiviruses carrying the miR-15a-16-1, miR-497-195 and their corresponding mutants to infect MDA-MB-231 cells, respectively. Three days post infection, we determined endogenous FASN expression of these cells by Western blot analyses. Compared to the two mutant miRNAs, miR-15a-16-1 markedly reduced endogenous FASN expression, but miR-497-195 only resulted in a marginal FASN decrease (Figure 3A). Bcl-2, a previously reported target of miR-15a-16-1, showed reduced expression in response to miR-15a-16-1, but not to miR-497-195. We then investigated how these miRNAs affect cell proliferation. In WST-1 assays using MDA-MB-231 cells, only miR-15a-16-1 significantly inhibited cell proliferation, whereas miR-497-195 was indistinguishable from the two control miRNA constructs (Figure 3B). Based on these data, our results indicate that miR-15a-16-1, but not miR-497-195, could both reduce endogenous FASN expression and inhibit breast cancer cell proliferation. Thus, in the following studies, we focused on exploring miR-15a-16-1-mediated FASN expression in breast cancer cells.

To determine which miRNA may contribute more in suppressing cell growth, we individually tested the effects of miR-15a and miR-16-1 on breast cancer cell proliferation. We infected MDA-MB-231 cells by lentiviruses expressing either miR-15a-16-1, miR-m15a-16-1, miR-15a-m16-1 or miR-m15a-m16-1, and then determined cell proliferation by WST-1 assays. As shown in Figure 3C, mutation of either miR-15a or miR-16-1 resulted in reduced inhibitory effects of miR-15a-16-1 on MDA-MB-231 cell proliferation. Consistently, both miR-m15a-16-1 and miR-15a-m16-1 retained partial inhibition to FASN expression in MDA-MB-231 cells compared to miR-15a-16-1 (Figure 3D). Meanwhile, a shRNA construct for FASN, shFASN, could more efficiently diminish FASN expression than miR-15a-16-1 (Figure 3D). In control experiments, shFASN also reduced cell proliferation than shCont, while miR-m15a-m16-1 could slightly attenuate cell proliferation compared to an empty vector (Supplementary Figure 1), suggesting that these mutant microRNAs exerted some adverse effects on cell growth. Overall, our data suggest that both miR-15a and miR-16-1 contribute to inhibiting FASN expression and breast cancer cell proliferation.

### FASN expression is primarily downregulated by miRNAs in mammary cells

**Figure 4:**
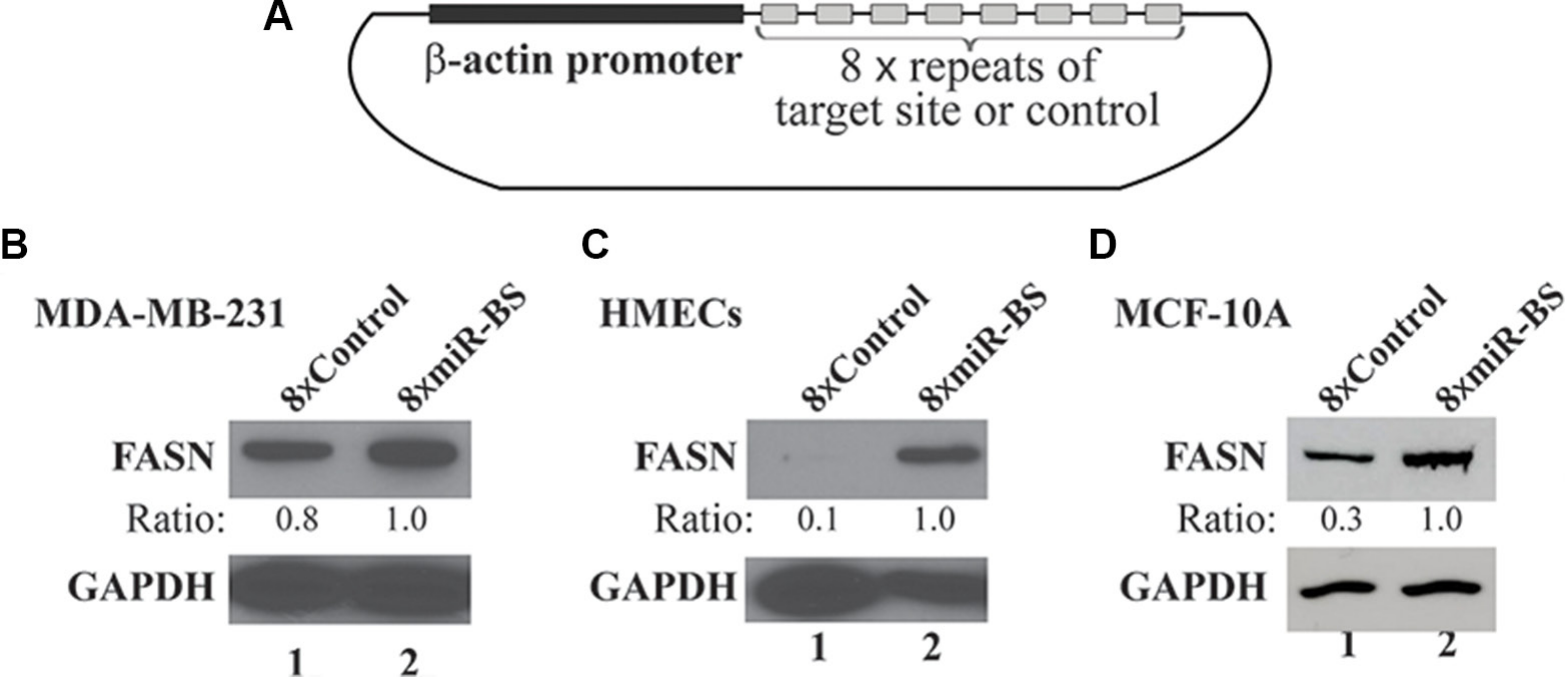
Effects of a sponge vector consisting of the miRNA binding site of the FASN 3′-UTR on endogenous FASN expression (**A**) Schematic structure of the sponge vector consisting of 8 repeats of the miR-15a-16-1 target site present in the FASN 3′-UTR. The plasmid is based on pSL5 lentiviral vector and the expression is driven by the β-actin promoter. (**B**) (**C**) and (**D**) Effects of the sponge vector on endogenous FASN expression. MDA-MB-231 cells (B), HMECs (C) and MCF-10A cells (D) were infected by lentiviruses for the sponge vector (8 × miR-BS; BS: binding site) and the control (8 × Control). Two days post infection, cells were cultured in medium containing 1 μg/ml of puromycin for another 3 days. The cells were collected and analyzed by Western blot analyses using the indicated antibodies.

To determine whether miR-15a-16-1-mediated repression plays a major role in regulating FASN expression in mammary cells, we generated a lentiviral sponge construct with eight repeats of a sequence containing the miR-15a-16-1 binding site on the FASN 3′-UTR driven by a chicken β-actin promoter (Figure 4A). As a control, we created a similar lentiviral vector with eight repeats of a scrambled sequence. We used these lentiviruses to individually infect MDA-MB-231 cells expressing high levels of FASN, and MCF-10A or normal HMECs expressing relatively low or undetectable FASN. The sponge vector 8xmiR-BS modestly increased FASN expression in MDA-MB-231 cells, but markedly elevated FASN expression in MCF-10A cells and HMECs, in comparison to the control vector (8xControl) (Figure 4B and 4C). Meanwhile, 8xmiR-BS also increased expression of Bcl-2 and YAP1, but not cyclin D1 in MCF-10A cells (Supplementary Figure 2A), although they are all reported targets of miR-15a and miR-16-1 [18, 29]. However, we did not observe significant changes in proliferation of MCF-10A cells expressing 8xmiR-BS (Supplementary Figure 2B). Overall, our data suggest that miR-15a-16-1 is a key regulator of FASN expression in mammary cells.

### Restored FASN expression in breast cancer cells partially rescues miR-15a-16-1-mediated cell growth inhibition

**Figure 5:**
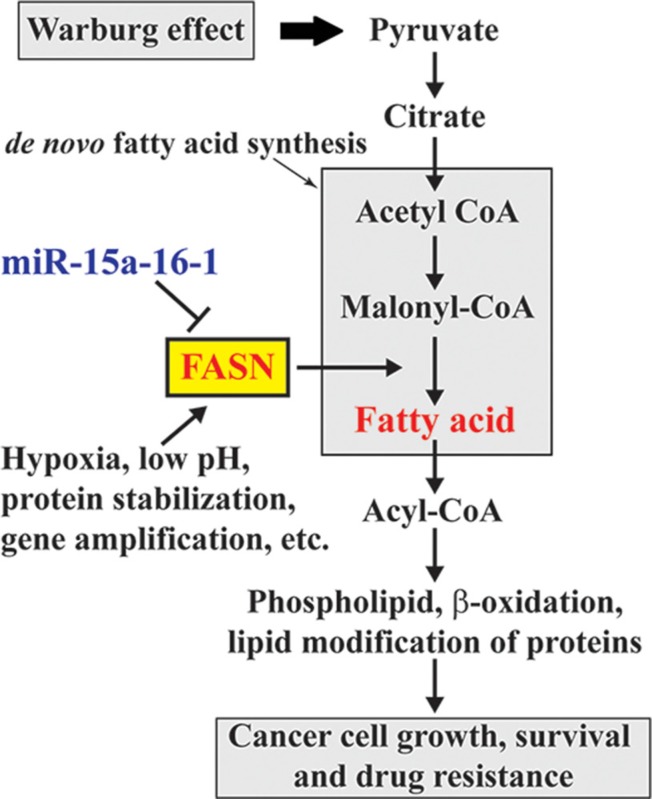
Restoration of miRNA-inhibited FASN expression and its effect on breast cancer cell proliferation MDA-MB-231 cells infected by miRNA-expressing lentiviruses or co-infected by FASN expressing lentivirus were collected for Western blot analyses to determine FASN expression (**A**) and WST-1 assays to test cell proliferation (**B**). Results are presented as a mean of three observations ± S.D. Statistical significance is shown by * indicating a *p* value < 0.05.

Each miRNA inhibits the expression of multiple mRNAs. If FASN is a primary target of miR-15a-16-1, restoration of FASN should rescue the proliferative retardation caused by ectopic miR-15a-16-1 expression. A lentivirus carrying FASN expression cassette without its 3′-UTR was used to infect MDA-MB-231 cells that were already infected by either miR-15a-16-1 or mutant miR-m15a-m16-1. As shown in Figure 5A, FASN expressing lentivirus successfully restored FASN protein levels downregulated by miR-15a-16-1, although it did not significantly further increase FASN levels in miR-m15a-m16-1-infected cells. We then determined whether restored FASN expression could rescue cell proliferative retardation caused by miR-15a-16-1. MDA-MB-231 cells expressing both miR-15a-16-1 and exogenous FASN showed better proliferation than these carrying only miR-15a-16-1, but still did not proliferate as well as the control group with miR-m15a-m16-1 (Figure 5B). Interestingly, ectopic FASN in MDA-MB-231 cells expressing miR-m15a-m16-1 did not further increase cell proliferation. The data suggest that FASN expression is an important phenotypic event associated with miR-15a-16-1, although these two miRNAs regulate multiple targets involved in cell proliferation.

## DISCUSSION

Deregulated gene expression contributes to mammary oncogenesis. Aberrant DNA methylation and histone modifications tangle the expression of genes involved in oncogenesis and thus promote breast cancer development and progression. Recently, accumulating data revealed the function of miRNAs in regulating expression of cancer-related genes and their roles in oncogenesis. Meanwhile, alterations of miRNA profiles in cancers and their potential in cancer diagnosis and prognosis has been frequently reported. To date, the biological function of miRNAs is mostly restricted to mediating gene expression, and their ultimate roles in oncogenesis depend on the activity of target genes. Multiple studies indicated that FASN is a potential prognostic marker and therapeutic target of cancers, including breast cancer [30, 31], while miR-15a-16-1 and miR-497-195 play a tumor suppressive role. We thus checked the 3′-UTR of FASN and discovered a potential target site of these two miRNA clusters in it. However, although all these miRNAs suppressed FASN 3′-UTR-mediated expression in reporter assays, only ectopic expression of miR-15a-16-1, but not miR-497-195, inhibited the endogenous FASN expression and reduced breast cancer cell proliferation.

Pairing between a microRNA 6–8 nt long seed sequence and a target sequence on a mRNA is the most important feature for different algorithms of miRNA target site prediction, but the overall duplex thermodynamic stability is also important for target silencing; thus, the same seed sequence does not guarantee comparable silencing efficiencies [32]. Whether a microRNA can target the mRNA needs experimental confirmation. Reporter assays entail constitutive misexpression of a reporter and overexpression of a miRNA, leading to their non-physiological interactions [33], and thus do not always represent endogenous scenario. As we previously reported, miR-26a markedly reduced the luciferase activity of an Ezh2 3′-UTR reporter, but showed no effect on endogenous Ezh2 expression in prostate cancer cells [28]. In the current study, we made the same observation for the miR-497-195, which inhibited the FASN 3′-UTR reporter but did not reduce endogenous FASN levels. Thus, whether a miRNA can target a gene will need experiments for its effect on the endogenous gene, as reporter assays may provide artificial results.

**Figure 6:**
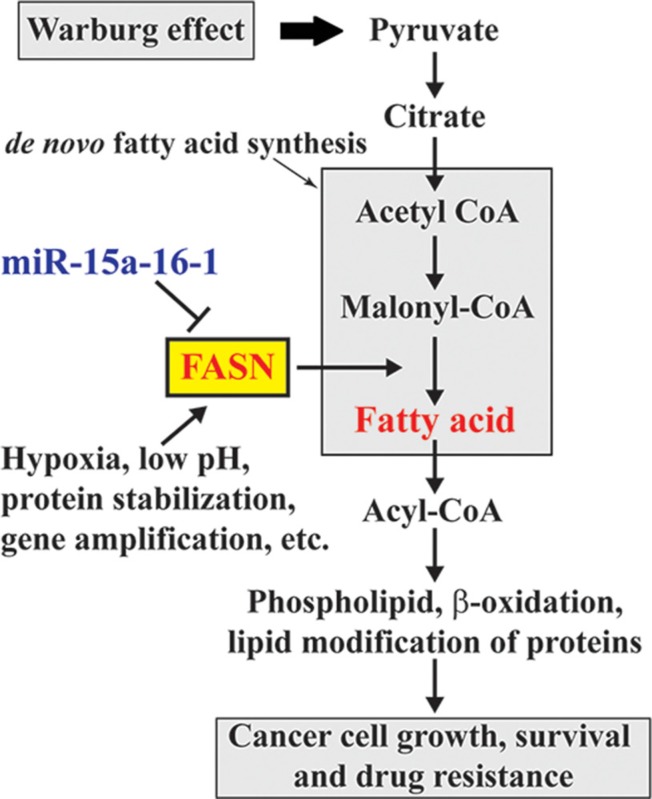
Schematic diagram for the involvement of miR-15a-16-1 regulated FASN expression in the Warburg effect of cancer cells miR-15a-16-1 inhibits FASN expression and in turn reduces fatty acid synthesis. This attenuates glucose utilization through glycolysis as a cellular resource in cancer cells.

FASN is overexpressed in cancers and plays an oncogenic role in cancer development and progression [1]. According to the Warburg effect, increased anaerobic glycolysis in cancer cells produces excess pyruvate, some of which can be converted to acetyl-CoA and used for *de novo* fatty acid synthesis. Elevated FASN promotes *de novo* fatty acid synthesis and leads to fatty acid accumulation, which alters various fundamental cellular process, including gene expression and signaling transduction, and ultimately promotes oncogenesis (Figure 6). Elevated FASN expression is mediated by multiple mechanisms. In this study, we demonstrated the negative regulation of FASN by miR-15a-16-1. Our data for the first time suggest that miR-15a-16-1 may suppress oncogenesis through blocking the downstream steps of the Warburg effect (Figure 6).

We speculate that altered expression of miRNAs is one important mechanism by which FASN expression is deregulated in breast cancer. To date, several miRNAs have been reported to inhibit FASN expression, including miR-142-3p, miR-320, miR-424 and miR-195 [23–26], but these studies were only carried out in osteosarcoma. Consistently, all these miRNAs have been shown to exhibit reduced expression in cancers and inhibit tumor cell proliferation or metastasis. However, it is still undetermined which of these miRNAs acts as a key regulator in downregulating FASN expression and inhibiting proliferation in mammary cells. In this study, we demonstrated that miR-15a-16-1 specifically inhibited FASN expression; more importantly, a sponge construct expressing 8 copies of the miR-15a-16-1 target sequence in the FASN 3′-UTR could markedly increase FASN expression in nontumorigenic MCF-10A cells and HMECs that typically show low or undetectable FASN protein (Figure 4C and 4D). This strongly suggests that FASN in mammary cells is downregulated by microRNAs that target the sequence used in generating our 8xmiR-BS sponge vector. Since our data indicated that miR-15a-16-1, but not miR-497-195, inhibits endogenous FASN expression (Figure 3A and 3B), it is logical to conclude that miR-15a and miR-16-1 are the key miRNAs responsible for suppressing FASN expression in normal mammary cells. The inconsistency for the activity of miR-195 in regulating FASN expression between our study and a previous report [25] is likely due to some subtle difference between breast cancer and osteosarcoma, such as difference in cellular protein composition and microenvironment.

Restoring FASN expression that was reduced by miR-15a-16-1 did not fully rescue the retarded cell proliferation (Figure 5B). This is not a surprising result because a miRNA may target hundreds of genes [12], some of which can contribute to cell proliferation. Many genes with oncogenic or proliferative activities have been reportedly targeted by miR-15a and miR-16-1, such as Bcl-2, cyclin D1, WNT3A, YAP1 and WT1 [18, 29, 34]. We observed that using a sponge vector 8xmiR-BS to inhibit miR-15a-16-1 only slightly increase YAP1 and Bcl-2, but did not alter cyclin D1. This may be attributed by binding preference of the microRNAs and difference in cellular contact. Meanwhile, the sponge vector did not enhance proliferation of MCF-10A cells, despite increased FASN expression. This suggests that MCF-10A, as an immortalized cell line, did not response sensitively to elevated FASN levels. In our experiments, lentivirus-infected HMECs did not grow well in WST-1 assays, likely due to its susceptibility to senescence. Some of these genes may play a role in mammary oncogenesis; thus, restoration of FASN expression only partially rescues proliferation of breast cancer cells. It is interesting to note that the co-infection of MDA-MB-231 cells by lentiviruses expressing FASN and miR-m15a-m16-1 did not markedly further increase FASN expression in MDA-MB-231 cells. This suggests that other mechanism may play a role in maintaining the equilibrium of FASN levels in breast cancer cells.

MiR-15a and miR-16-1 are downregulated in different cancers and target multiple oncogenic or proliferative genes. Meanwhile, FASN has been proposed as a bona fide therapeutic target for cancers and many FASN inhibitors were developed as potential anticancer drugs [35, 36]. In this study, we provided strong evidence to show that miR-15a-16-1 targets FASN and reduces breast cancer cell proliferation. Our data implicate the involvement of miR-15a-16-1 in the Warburg effect and reinforce the notion for the potential of miR-15a and miR-16-1 as anticancer therapeutics in breast cancer treatment.

## MATERIALS AND METHODS

### Cell culture, transient transfection and lentiviral production

Tumorigenic MDA-MB-231 and MCF-7 cell lines were obtained from the American Type Culture Collection (Manassas, VA) and cultured as recommended by the manufacturer. Normal human mammary epithelial cells (HMECs) were generously provided by Dr. Martha R. Stampfer at Lawrence Berkeley National Laboratory, Berkeley and cultured as previously described [37]. Lipofectamine 2000 was used in transient transfection based on the protocol provided by the manufacturer. The production of lentivirus followed our previously reported protocol [38]. Briefly, 293T cells were transfected with a miRNA or sponge expressing lentiviral vector, together with three packaging plasmids (pMDLg/pRRE, pRSVRSE and pVSV-G) using a polyethylenimine (PEI)-based transfection protocol [39]. Medium containing viral particles was collected 48 h after transfection. To infect cells, lentivirus-containing medium was added to the cells and supplied with 8 μg/ml polybrene. The medium was replaced with normal culture medium 6 h post viral addition.

### Antibodies and reagents

Antibodies, catalog numbers and their vendors include: FASN (cat# 610963, BD Transduction Labs), Bcl-2 (cat# 2870, Cell Signaling Technology), cyclin D1 (cat# 2978, Cell Signaling Technology), YAP1 (cat# 8418, Cell Signaling Technology) and GAPDH (cat# 10R-G109A, Fitzgerald Industries International). Lipofectamin 2000 (Invitrogen/Thermo Fisher Scientific) was used as a reagent for transient transfections. Polyethylenimine (PEI, branched) (cat# 408727, Sigma-Aldrich) was used in transfection to produce lentiviruses. Oligonucleotides for PCR and DNA sequencing were synthesized by the Integrated DNA Technologies, Inc.

### Construction of miRNA, shRNA and sponge expression plasmids

The generation of microRNA expression plasmids for miR-15a-16-1 and miR-497-195 using their corresponding genomic DNA sequences was based on a previously published protocol [40]. We performed “nested PCR” using the genomic DNA from HMECs as a template to amplify the miR-15a-16-1 and miR-497-195 sequences with the following primers: for miR-15a-16-1, forward: TGTGAAGTCTTACCTATTTGATGA, reverse: GTGTTTCCTTAGCTATCCAATGAG; for miR-497-195, forward: CAGTCATTCCCTATTTCTTCTGCC, reverse: ACTGTTCATGCCGGACCTGTGG. The resultant products were subjected to another PCR using primers with incorporated BamHI and EcoRI restriction enzyme sites (underlined): for miR-15a-16-1, forward: CGAGGGATCCGATCCCCTGAGCTGAGTTCCTAC, reverse: CACGGAATTCGGGGCTGCCACCTCGCTA TTCC, and for miR-497-195, forward: CGAGGGATCCCC TCATTTTATTCTTTGTGTTTCC, reverse: CACGGAAT TCAAATTCCTCTAATGCTGCATAAGC. The PCR products (707 and 830 base pairs (bps), respectively) were digested by BamHI and EcoRI, followed by subcloning into a lentiviral vector pSL4 digested by the same restriction enzymes to obtain pSL4-miR-15a-16-1 and pSL4-miR-497-195 lentiviral vectors.

To generate lentiviral constructs expressing the mutated forms of these miRNAs, we individually or combinatorially replaced their seed sequences (AGCAGCA) by sequences containing an ApaLI site (GTGCAC) or a MluI site (ACGCGT). The mutated seed sequences are: for miR-15a: GTGCACA; for miR-16-1: ACGCGTA; for miR-497: GTGCACA; for miR-195: AACGCGT. Thus, with their combinations, we generated pSL4 lentiviral vectors for the expression of miR-m15a-16-1, miR-15a-m16-1, miR-m15a-m16-1, miR-m497-195, miR-497-m195, and miR-m497-m195 (m: mutated).

A lentiviral vector expressing FASN-targeting shRNA, shFASN, was constructed using our previously reported protocol [38, 41]. The target sequence on the FASN cDNA is GCACGGTCGCTTCCTGGAAATT. A scrambled sequence GGGACTACTCTATTACGTCATT was used to generate a control shRNA, shCont.

To generate a sponge construct expressing 8 repeats of a sequence embracing the miR-15a-16-1 target site on the FASN 3′-UTR (AGAAATGATTCAAATTGCTGCTT; the binding site for the seed sequence is underlined), we take the advantage of XbaI and SpeI as isoschizomers to carry out digestion and ligation repetitively. The two initial oligonucleotides to be annealed are: GATCC CAA tctaga AGAAATGATTCAAATTGCTGCTT actagt CAA G, and AATTC TTG actagt AAGCAGCAATTTGAATCATTTCT tctaga TTG G (after annealing, the underlined uppercases form two cohesive ends for BamHI and EcoRI, respectively; the underlined lowercases are the XbaI/TCTAGA and SpeI/ACTAGT sites). The annealed products were digested by SpeI and XbaI, respectively, and the two digested products were simultaneously ligated into a lentiviral vector pSL5 [42] cleaved by BamHI and EcoRI to create a vector of 2xmiR-BS (BS: binding site) driven by the chicken β-actin promoter. This plasmid was digested by BamHI+SpeI and XbaI+EcoRI, respectively, and the short fragments were gel-purified followed by the same ligation as above to generate a vector of 4xmiR-BS. A repeated procedure led to the production of the sponge vector 8xmiR-BS. A control vector, 8xControl, was also generated following the same procedure and a scrambled sequence (TCCGGATGTTATAATGCAGCAA) was used to replace the miRNA target site. The two initially annealed oligonucleotides are: GATCC CAA tctaga TCCGGATGTTATAATGCAGCAA actagt CAA G and AATTC TTG actagt TTGCTGCATTATAACATCCGGA tctaga TTG G.

### Reporter assay

In wt reporter, an 899-bp fragment containing the last 67 bps of the FASN coding region and the entire > 800-nucleotide sequence of the FASN 3′-UTR is subcloned downstream of Gaussia luciferase (Gluc) driven by the PGK promoter. To mutate the putative target site (TTGCTGCT) for miR-15a-16-1 and miR-497-195, a sequence containing a NcoI site (TCCATGGT) was used to replace it.

In a reporter assay, each well of MCF-7 cells cultured on a 24-well plate was transfected with 50 ng of a wt reporter or mutated reporter plasmid, different amounts of expression plasmid for miRNAs or their mutants, and 100 ng of plasmid expressing secreted alkaline phosphatase (SEAP) driven by the β-actin promoter. A control miRNA (miR-m15a-m16-1 or miR-m497-m195) was used to compensate the miRNA amounts to achieve an equal amount of transfected DNA in each well. Aliquots of medium from the transfected wells were collected 48 h posttransfection to measure luciferase activity. Fifty μl of the medium (diluted if necessary) was mixed with 100 μl of substrate solution containing 0.5 μg/ml of coelenterazine (CTZ), 200 mM NaCl, 50 mM Tris·HCl and 0.01% Triton X-100, at pH 8.7. The light emission was measured at a wavelength of 480 nm and normalized with the SEAP expression [43].

### WST-1 cell proliferation assay

MDA-MB-231 cells were infected by Lentivirus and puromycin-selected as needed in the experiments. These cells were then plated at a density of 3,000 cells/well in 96-well plates. At each time point, cell proliferation in triplicates was measured using WST-1 (Roche) following the manufacturer's protocol.

## SUPPLEMENTARY MATERIALS


