# Supplementary material for: Fatty acid synthase is a primary target of MiR-15a and MiR-16-1 in breast cancer
Source: Oncotarget. 2016 Oct 5;7(48):78566–76. doi: 10.18632/oncotarget.12479 (PMC5346660; doi:10.18632/oncotarget.12479)
Supplement: Supplementary file 1 [file oncotarget-07-78566-s001.pdf]

Fatty acid synthase is a primary target of MiR-15a and MiR-16-1 in breast cancer

Supplementary Materials

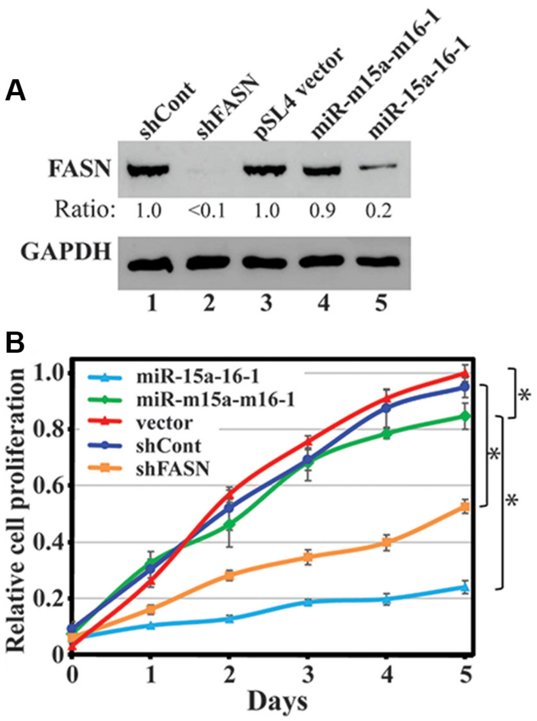

Supplementary Figure S1: Effects of lentivirus and FASN knockdown on breast cancer cell proliferation. MDA-MB-231 cells infected by lentiviruses of an empty vector or carrying a control shRNA (shCont), FASN shRNA (shFASN) or miRNAs as labeled were collected for Western blot analyses to determine FASN expression (**A**) and WST-1 assays to test cell proliferation (**B**). Results are presented as a mean of three observations ± S.D. Statistical significance is shown by *indicating a p value < 0.05.

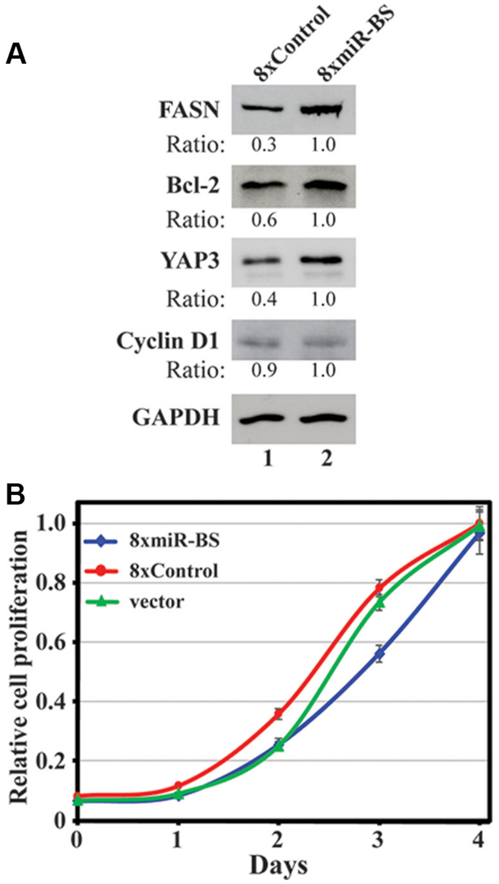

Supplementary Figure S2: Effects of microRNA sponge vector on gene expression and MCF-10A cell proliferation. MCF-10A cells were infected by lentiviruses carrying 8 × Control and 8xmiR-BS, followed by puromycin selection. Three days post selection, fractions of cells were collected for Western blot analyses using antibodies as indicated (**A**) and other fractions of cells, together with these infected by an empty vector, were tested for cell proliferation using WST-1 assays.
